# Prevalence and characteristics associated with diabetes mellitus and impaired fasting glucose among people aged 15 to 64 years in rural and urban Rwanda: secondary data analysis of World Health Organization surveillance data

**DOI:** 10.11604/pamj.2022.41.115.30682

**Published:** 2022-02-09

**Authors:** Charlotte Munganyinka Bavuma, Jean Berchmans Niyibizi, Leopold Bitunguhari, Sanctus Musafiri, Ruth McQuillan, Sarah Wild

**Affiliations:** 1 Kigali University Teaching Hospital, School of Medicine and Pharmacy, College of Medicine and Health Sciences, University of Rwanda, Kigali, Rwanda,; 2 Single Project Implementation Unit, College of Medicine and Health Sciences, University of Rwanda, Kigali, Rwanda,; 3 Usher Institute, University of Edinburgh, Scotland, United Kingdom

**Keywords:** Diabetes, rural, Rwanda

## Abstract

**Introduction:**

diabetes mellitus is an increasing public health burden in developing countries. The magnitude of diabetes association with traditional risk factors for diabetes have been given less attention in rural population. This study aims to determine the prevalence of diabetes and impaired fasting glucose and to assess associated characteristics to hyperglycemia in rural and urban Rwanda.

**Methods:**

this is a secondary analysis of data from a population-based cross-sectional study of 7240 people describing risk factors for non-communicable diseases using the WHO stepwise methods (STEPS). Relative frequencies of variables of interest were compared in rural and urban residence using Pearson chi-square tests. Diabetes and impaired fasting glucose were combined in a single hyperglycemia variable and odds ratios with 95% confidence intervals were used to explore associations between hyperglycemia, socio-demographic and health factors in urban and rural populations.

**Results:**

the prevalence in rural and urban areas was 7.5% and 9.7% (p.005) for diabetes and 5.0% and 6.2% for impaired fasting glucose (p.079) respectively. Obesity (AOR 2.57: CI: 0.86-7.9), high total cholesterol (AOR 3.83: CI: 2.03-7.208), hypertension (AOR 1.18: CI: 0.69-2.00), increasing age were associated with hyperglycemia in urban participants but only high total cholesterol and low high density lipoproteins (HDL) cholesterol were risk factors for hyperglycemia in rural participants.

**Conclusion:**

approximately one in six people in Rwanda have hyperglycemia. The magnitude of the association with traditional risk factors for diabetes differ in rural and urban settings. Different approaches to primary and secondary prevention of diabetes may be needed in rural populations.

## Introduction

Diabetes mellitus (DM) has become a global public health challenge that is particularly on the increase in low and middle income countries (LMIC) [1]. The numbers of people with DM are expected to rise from 425 million in 2017 to 629 million by 2045 worldwide and to increase by threefold in LMIC [1], however the factors driving this rapid increase are not well understood. The rapid urbanization in LMIC, adoption of sedentary lifestyles, changes in diet and increasing prevalence of obesity are reported to be the major determinants of the increasing prevalence of diabetes [2]. The current frameworks for understanding Chronic noncommunicable diseases (NCDs) link diabetes to rapid development and modernity; however, these rely on global aggregate data, which do not allow for a more nuanced analysis of determinants in remote rural areas in LMICs [3]. Furthermore, emerging evidence shows that type 2 diabetes is related to socio-economic status, often with a positive association in developing countries but with an inverse association in developed countries [4-8]. While the burden caused by NCDs is growing at alarming pace, the challenge to tackle infectious diseases, even before the Covid-19 pandemic, remains. Chronic infectious diseases such as Human Immunodeficiency Virus (HIV) infection and its treatment might contribute to the increasing number of people with diabetes in LMIC and uncontrolled diabetes can make control of chronic infections such as tuberculosis even more difficult [9]. Type 2 diabetes is reported to contribute over 90% of all diabetes cases in sub-Saharan Africa whereas type 1 diabetes, gestational diabetes, and variant forms such as atypical ‘ketosis-prone´ diabetes and malnutrition-related diabetes constitute the remainder [10].

These figures are mostly from urban settings, while evidence shows that diabetes prevalence is also increasing markedly in rural areas of LMIC where the distribution of different types of diabetes may differ [11]. In our recent descriptive study characterizing diabetes in rural Rwanda, we found that more than half the people with diabetes were classified as type 1 diabetes based on clinical features, including early insulin treatment requirement [12]. This apparent overrepresentation of type 1 diabetes might be due to the presence of atypical diabetes that overlap with the type 1 diabetes phenotype [12]. Currently in Rwanda, there are significant efforts to reduce the prevalence of traditional risk factors for diabetes and cardiovascular diseases. For example population-based interventions such as the “car free day” and “friday afternoon physical activity” sessions for public servants program have been established to raise the awareness of the benefits of doing physical exercise. Although interventions targeting the reduction of obesity and physical inactivity play a key role in decreasing cardiovascular diseases and diabetes, they may not be effective in settings where traditional risk factors are not major contributors to the increasing prevalence of diabetes. There is limited information on risk factors for hyperglycemia in rural populations in Rwanda. Given that the majority of Rwandan residents live in rural areas (80.9% in 2019 according to the 2019-2020 Rwanda demographic and health survey), it is important to understand the role of traditional risk factors for diabetes in rural areas to inform future research and contextualized preventive strategies. The aim of this study is to determine the prevalence of diabetes and impaired fasting blood glucose in rural and urban Rwanda and to describe the factors associated with hyperglycemia using the data from WHO steps survey.

## Methods

**Study design:** this is a secondary analysis of cross-sectional data collected during the NCD risk factors screening survey in Rwanda. The survey was conducted in 2012 using the WHO steps survey approach through three main steps: participants interview to collect socio-demographic data and known health conditions such as diabetes and hypertension, physical measurement including height, weight and waist circumference and laboratory tests (fasting blood glucose, total cholesterol and high density lipoproteins (HDL) cholesterol.

**Sample size and method:** the sample size was calculated using the formula:


$$N=Z^{2}P\left(1-P\right)/e^{2}$$

and assumptions described by WHO STEPS survey approach to non-communicable disease risk factor surveillance [13,14], where N= sample size, Z= level of confidence, P= baseline level of the selected indicator and e= margin of error. P was estimated at 0.50 (recommended by the steps survey guidelines when the estimated baseline is unknown), Z= 1.96 (at 95% confidence interval), e= 0.05, thus the estimated sample size was:


$$N=1.96^{2}\times 0.5\left(1-0.5\right)/0.05^{2}=384$$

This basic sample size was adjusted for design effect for complex sample design (1.50), age-sex estimates, 15-64 age range (10 year intervals) and the required sample size was therefore:


N=384×1.5×10=5760

Assuming a non-response rate of 20%, the final minimum sample size was therefore adjusted upward to: 5760/0.8 = 7200. A total of 7240 participants were enrolled using a multistage cluster sampling strategy to select a nationally representative sample. Rwanda is divided into 30 districts which are in turn divided into sectors making up a total of 416-sectors. Each sector is sub-divided into cells and then into villages. In the first stage all 30 districts constituted strata and therefore were all sampled. In the second stage villages were randomly selected from sectors using a probability proportional to size sampling. Overall 180 (1.2%) villages were selected from a total of 14837 in the country. From each village 40 households were randomly selected based on the list provided by the National Institute of Statistics of Rwanda. Eligible participants were aged from 15-64 years and were selected using Kish sampling method [15]. The data collection was performed by 16 trained teams of three members composed of one supervisor, one lab technician and one epidemiologist between November 2012 and March 2013 [15].

**Statistical analysis:** the major outcome for this analysis was hyperglycemia defined as any of impaired fasting blood glucose (fasting blood glucose between 110-126mg/dl (5.6-6.1mmol/L) and diabetes (fasting blood glucose ≥ 126mg/dl (6.1mmol/L) and self-reported diabetes on treatment). The following variables were included in the analysis as potential risk factors for hyperglycemia status: age, sex, residence province, marital status, education level, body mass index (categorized as underweight for BMI (in kg/m^2^) <18.5, normal for BMI ≥ 18.5 and <25, overweight for BMI ≥25 and <30, obesity ≥30) smoking status, fruit and vegetable consumption, hypertension status, alcohol consumption, HDL cholesterol and total cholesterol. Using STATA version 13, the descriptive analysis was conducted by cross-tabulating each variable with area of residence (rural and urban) and results were presented in the form of relative frequencies. The Pearson chi-square test at 5% level of significance was computed. The bivariate analysis was performed to describe associations between hyperglycemia (dependent variable) and each independent variable to generate crude odds ratio (OR) and 95% confidential intervals (CI). Factors found to be statistically significantly associated with hyperglycemia (p≥0.05) were included in the full multivariable model. Variables identified as being independently associated with hyperglycemia were those whose p values were equal or lesser than the level of significance (0.05) in the final multivariable logistic regression models. All analyses were conducted separately for rural and urban populations.

**Ethical consideration:** primary data collection received ethical clearance from the Rwanda national Ethics Committee. Approval to use this dataset for secondary analysis was granted by the Rwanda Biomedical Centre.

## Results

This study included a total of 7240 respondents, of whom 37.2% were men, 78.3% lived in rural areas, 14.7% were overweight, 3.5% were obese, 84% had never smoked, 47.4% had low high density lipoprotein cholesterol (HDL), 3.7% had high total cholesterol, 1.7% had impaired fasting glucose and 3.2% had diabetes. There was no significant difference in the prevalence of diabetes between men and women in both rural and urban settings. As indicated in Table 1, the prevalence of diabetes was higher in urban than in rural settings; however, the prevalence of impaired fasting glucose was similar in both settings. There were similarly large proportions of women in rural and urban area. The prevalence of low education level was high in both settings, but a hgher proportion of urban than rural dwellers reported higher education level. Moreover obesity and overweight, greater waste to height ratio, high total cholesterol level, and HIV infection were more prevalent in urban than rural settings see Table 1. The bivariate analyses reported in Table 2 showed that high waist to height ratio, low HDL cholesterol and high total cholesterol are significantly associated with hyperglycemia in rural and urban settings. Increasing age, overweight and obesity and hypertension were significantly associated with hyperglycemia in urban areas only. Furthermore, prevalence of hyperglycemia was higher in rural Eastern and Southern provinces than Western and Northern rural provinces, and in Kigali City and urban Eastern province than other urban areas. The multivariable logistic regression for rural areas (Table 3) showed that rural Western and Northern provinces´ residents had lower odds of hyperglycemia (AOR: 0.45, 95% CI: 0.31-0.66, and AOR: 0.30, 95% CI: 0.18-0.50 respectively p value < 0.001). HDL (AOR: 0.53, 95% CI: 0.40-0.71, p value < 0.001) was inversely associated with hyperglycemia. The odds of having hyperglycemia was high among respondents with high total cholesterol compared to those with cholesterol <5.17 mmol/l. In urban settings, the multivariable logistic regression (Table 4) showed that increasing age was associated with a linear increase in the risk of having hyperglycemia. Residents in urban Eastern Western, Northern and Southern provinces had lower odds of hyperglycemia compared to Kigali City. Respondents with high HDL cholesterol were less likely to have hyperglycemia. The odds of having hyperglycemia was almost 4 times higher among respondents with high total cholesterol ≥ 5.17 mmol/l compared to those with low total cholesterol < 5.17 mmol/l. The odds of having hyperglycemia was slightly higher for participants with hypertension compared to people who are free of hypertension.

**Table 1:** demographic, lifestyle and health characteristics of participants in the Rwanda NCDs risk factors survey, 2012

Variable	Rural		Urban		P-value
	N	%	N	%	
**Sex**	5,668		1,572		0.082
Male		37.7		35.3	
Female		62.3		64.7	
**Age**	5,668		1,572		0.000
15-24		19.6		25.7	
25-34		32.7		34.4	
35-44		21.8		19.9	
45-54		15.4		11.9	
≥55		10.5		8.1	
**Education level**	5,656		1,572		0.000
Low level of education		89.4		71.8	
High level of education		10.6		27.9	
Refused to answer		0.0		0.3	
**BMI**	5,609		1,562		0.000
Lean		7.3		7.4	
Normal		76.0		62.5	
Obese & overweight		16.7		30.1	
**Waist_height ratio**	5,668		1,572		0.000
Ratio <0.5		66.2		56.6	
Ratio ≥0.5		33.8		43.4	
**HDL**	5,668		1,572		0.167
Low HDL (< 1.3 mmol/l)		25.7		27.4	
High HDL (≥ 1.3 mmol/l)		74.3		72.5	
**Cholesterol**	5,668		1,572		0.000
<5.17mmol/l		95.4		91.5	
≥5.17mmol/l		4.6		8.5	
**Duration on ARV**	136		72		0.875
<60 months		69.1		68.1	
≥60 months		30.9		31.9	
**HIV status**	4,430		1,278		0.000
Positive		3.4		6.7	
Negative		96.6		93.3	
**Alcohol consumption**	5,668		1,572		0.036
Did not drink 5+ drinks		75.4		78.0	
Drank 5+ drinks at least once		24.6		22.0	
**Smoking status**	5,655		1,571		0.000
Never smoked		83.2		87.0	
Ever smoked		16.8		13.0	
Hypertension	5,668		1,572		0.065
No		83.0		81.0	
Yes		17.0		19.0	
**Fruit and vegetable consummation**	5,668		1,572		0.448
≥5 servings of fruit/veg on average per day		1.0		0.8	
<5 servings of fruit/veg on average per day		99.0		99.2	
**Impaired fasting blood glucose**	5523		1,518		0.079
No		95.0		93.8	
Yes		5.0		6.2	
**Reported and newly diagnosed diabetes**	5,668		1,572		0.005
No		92.5		90.3	
Yes		7.5		9.7	
**Hyperglycemia**	5247		1426		0.000
No		95.7		92.9	
Yes		4.3		7.1	

**Table 2:** bivariate association of hyperglycemia with socio-demographic, behavioral and metabolic risk factors

Variable	Rural				Urban			
	Hyperglycemia				Hyperglycemia			
	N	Yes (%)	Crude OR(95%CI)	P-value	N	Yes (%)	Crude OR(95%CI)	P-value
**Sex**				0.06				0.91
Male	2,137	4.9	1		555	7.2	1	
Female	3,531	3.9	0.77 (0.59- 1.01)		1,017	7.0	0.97(0.63-1.48)	
**Age**				0.16				
15-24	1,109	3.4	1		404	3.9	1	0.005*
25-34	1,85	3.9	1.15 (0.75-1.75)		541	6.	1.73(0.90-3.28	
35-44	1,238	4.3	1.29 (0.83-2.01)		313	6.9	1.82(0.90-3.67)	
45-54	874	5.1	1.53 (0.96- 2.43)		187	11.5	3.17(1.56-6.44)	
≥55	594	5.7	1.72 (1.06-2.84)		125	12.2	3.39(1.58-7.25)	
**Education level**								0.06
Low level of education	5,055	4.4	1	0.33	1,12	6.2	1	
High level of education	601	3.5	0.79 (0.49- 1.28)		438	9.2	1.52(0.99-2.33)	
Refused to answer	0	-	-		5	33.3	7.54(0.67-84.30)	
**BMI**				0.86				0.01*
Lean	412	3.8	1		115	4.8	1	
Normal	4,261	4.3	1.13(0.66- 1.94)		976	5.9	1.24(1.48-3.18)	
Obese and overweight	936	4.4	1.18(0.64- 2.18)		471	10.3	2.30(0.88-5.98)	
**Waist_height ratio**				0.04*				0.05*
Ratio <0.5	3,750	3.8	1		890	6.0	1	
Ratio ≥0.5	1,918	5.1	1.35 (1.02- 1.77)		682	8.6	1.49(1-2.23)	
**HDL**				<0.001*				<0.001*
Low HDL (< 1.3 mmol/l)	1,456	6.9	1		431	12.7	1	
High HDL (≥ 1.3 mmol/l)	4,212	3.4	0.48 (0.36- 0.63)		1,141	5.2	0.38 (0.25-0.57)	
**Cholesterol**				<0.001*				<0.001*
<5.17mmol/l	5,409	3.9	1		1,439	6.1	1	
≥ 5.17mmol/l	259	20.4	6.28(3.85-10.25)		133	26.0	5.45(3.08- 9.62)	
**HIV status**				0.6				0.062
Positive	152	3.5	1		86	13.0	1	
Negative	4,278	4.2	1.21 (0.49- 2.98)		1,192	19.1	0.48(0.24-98)	
**Alcohol consumption**				0.94				
Did not drink 5+ drinks	4,276	4.3	1		1,226	7.1	1	0.88
Drank 5+ drinks at least once	1,392	4.2	0.99(0.72- 1.35)		346	6.9	0.96(0.59-1.57)	
**Smoking status**				0.4				0.90
Never smoked	4,705	4.2	1		1,366	7.1	1	
Ever smoked	950	4.8	1.16 (0.82- 1.64)		205	6.9	0.96(0.52-1.76)	
**Hypertension**				0.28				0.02*
No	4,703	4.1	1		1,273	6.2	1	
Yes	965	4.9	1.21 (0.86-1.69)		299	10.4	1.73(1.10-2.72)	
**Province**				<0.001*				<0.001*
City of Kigali	-	-	-		824	9.6	1	
Eastern	1,589	6.0	1		124	9.9	1.02(0.49-2.13)	
Western	1,715	2.7	0.43(0.30- 0.63)		208	4.9	0.48(0.24-0.96)	
Northern	1,036	1.9	0.31(0.19- 0.51)		182	1.1	0.10(0.026-0.44)	
Southern	1,328	6.2	1.04 (0.75- 1.43)		234	4.4	0.42(0.21-0.84)	
**Fruit and vegetable consumption**				0.21				0.11
≥5 servings of fruit/vegetables per day	55	8.3	1		12	25.0	1	
<5 servings of fruit/vegetables per day	5,613	4.2	0.48 (0.17-1.36)		1,560	7.0	0.22(0.04-1.13)	

**Table 3:** risk factors associated with hyperglycemia in rural participants in the 2012 WHO steps study in Rwanda

Variables	Full model	
	Adjusted OR (95% CI)	P-value
Province		
**City of Kigali**	-	-
Eastern	1	
Western	0.45 (0.31-0.66)	<0.001*
Northern	0.30 (0.18-0.50)	<0.001*
Southern	1.08 (0.77-1.50)	0.662
**Waist / height ratio**		
Ratio <0.5	1	
Ratio ≥0.5	1.20 (0.90-1.59)	0.218
**HDL**		
Low HDL (< 1.3 mmol/l)	1	
High HDL (≥ 1.3 mmol/l)	0.53 (0.40-0.71)	<0.001*
**Cholesterol**		
<5.17mmol/l	1	
≥5.17mmol/l	4.70 (2.80-7.89)	<0.001*

**Table 4:** risk factors associated with hyperglycemia in urban participants in the 2012 Rwanda WHO STEPS survey

Variables	Full model		Adjusted model	
	Adjusted OR (95% CI)	P-value	Adjusted odds	P-value
**Age**			ratio(95% CI)	
15-24	1		1	
25-34	1.50(0.77- 2.93)	0.235	1.24(0.87-1.78)	0.230
35-44	1.60(0.77-3.36)	0.211	1.33(0.91-1.96)	0.14
45-54	2.70(1.25-5.84)	0.011*	1.75(1.18-2.62)	0.00*
55 and above	4.37(1.83-10.47)	0.001*	1.94(1.15-2.99)	0.00*
**Province of residence**			6.30(0.67-58.58)	0.10
City of Kigali	1		1	
Eastern	0.78(0.35-1.70)	0.529	0.59(0.42-0.83)	0.00*
Western	0.32(0.16-0.67)	0.002*	0.26(0.18-0.39)	<0.001*
Northern	0.09(0.02-0.39)	0.001*	0.15(0.09-0.26)	<0.001*
Southern	0.32(0.15-0.65)	0.002*		
**BMI category**			0.55(0.38-0.76)	<0.001*
Lean	1		1	
Normal	1.27(0.48-3.38)	0.630	-	-
Overweight or obese	2.57(0.86-7.69)	0.092		
**Waist to height ratio**			-	-
Ratio less than 0.5	1		1	
Ratio greater or equal to o.5	0.71(0.40-1.24)	0.224		
**HDL category**			-	-
HDL<1.3mmol/l	1		1	
HDL≥ 1.3 mmol/l	0.41(0.27-0.64)	<0.001*		
**Cholesterol category**			0.52(0.40-0.66)	<0.001*
Cholesterol<5.17mmol/l	1		1	
Cholesterol>5.17mmol/l	3.83(2.03-7.208)	<0.001*		
**Hypertension status**			4.52(3.06-6.65)	<0.001*
No	1		1	
Yes	1.18(0.69-2.00)	0.554		

## Discussion

The prevalence of diabetes of 3.2% in 15-64 year old corresponds to a population of 222,511 in need of care to reduce the risk of complications of diabetes and premature mortality. Diabetes is a chronic disease which requires lifetime treatment and for which complications create an economic burden on society [16,17]. Diabetes results in direct medical cost, direct nonmedical cost and indirect cost [18] thus, appropriate and contextualized policies and resources are required to provide care for people with diabetes in Rwanda. In this study, the prevalence of diabetes was higher in urban than rural participants, and the prevalence of Impaired fasting glycaemia (IFG) was similar in both settings. Differences in rural-urban prevalence of diabetes has been reported in other developing countries[19,20]. In some settings that difference might be explained by better access to diabetes screening and health services, which could contribute to higher prevalence of known diabetes in urban areas. IFG is a risk factor for overt diabetes and its high prevalence in rural and urban settings, highlights the importance of effective approaches to prevent diabetes.In both rural and urban settings, low HDL cholesterol and high total cholesterol were found to be related to hyperglycemia in our study and this is consistent with similar studies [21,22]. This is not surprising as low HDL cholesterol and high glucose are known to cluster together along with hypertension, hypertriglyceridemia, central obesity and insulin resistance in the metabolic syndrome [23]. A bidirectional relationship exists, as low HDL cholesterol level is associated with impaired beta-cell function leading to glucose deregulation [24] and hyperglycemia may decrease HDL cholesterol synthesis [25]. Previous studies have shown, consistent with our results, that total cholesterol is associated with uncontrolled diabetes [26,27]. Low HDL cholesterol belongs to a set of lipid profiles which increase the risk of diabetes and cardiovascular diseases [28]. An integrated approach to managing all cardiovascular risk factors is recommended by most recent guidelines in developed countries, such as the 2016 European guidelines on cardiovascular disease (CVD) prevention in clinical practice and American Diabetes Association guideline [29,30]. However, health facilities in remote areas are not able to analyze lipid profiles, even though dyslipidemia is a key risk factors for cardiovascular diseases

Our finding that older age is associated with hyperglycemia in urban individuals collaborates with other studies [31,32]. Interestingly, age was not associated with hyperglycemia in rural individuals. Possible explanations for this finding include survival bias, higher risk of atypical forms of diabetes related to childhood under nutrition among young adults in rural settings and potential for an interaction between age and physical inactivity in adults in urban settings [33]. Surprisingly, in our study, obesity was not found to be associated with hyperglycemia in the multivariable analysis in either rural or urban residents, but higher odds of hyperglycemia associated with obesity in urban dwellers were observed in bivariate analysis. Prevalence of obesity among participants in this study was low compared to some countries in the region, so the power to detect an association may be limited [34,35]. Type of diabetes was not specified in this study. This could contribute to the lack of association between obesity and hyperglycemia if there were relatively high proportions of people in the hyperglycemia group with type 1 or atypical diabetes for which the association with obesity is weaker or inverse. Obesity has been recognized as a risk factor for type 2 diabetes because of its association with insulin resistance [36]. In addition ethnic and sex differences in the association between obesity and diabetes that may be relevant to this study have been reported [37,38]. The implication of this finding is that prevention strategies and treatment guidelines should be adapted to local situations because key risk factors identified in some populations may not have the same relevance in other settings.

This study did not identify alcohol as an independent risk factor for hyperglycemia but other epidemiologic studies showed that drinking alcohol is associated with increased risk of diabetes [39], and its consumption is associated with risk of metabolic syndrome. Nonetheless alcohol consumed in moderation does not appear to increase the risk of type 2 diabetes and might have a protective effect [40] but this is controversial as there is now evidence suggesting that there is no safe lower limit for cardiovascular disease [41]. Living in the Eastern or Southern province was independently associated with higher odds of hyperglycemia among rural participants, and-living in Kigali City was associated with higher odds of hyperglycemia among urban participants. The Eastern province has been reported to have higher numbers of immigrants than other provinces, the reason of migration and spatial mobility being the rapid economic growth after 2002 [42]. However, it was not possible to distinguish native residents and migrants in this study, so it is not clear whether this is relevant to this study. Clearly both the disease patterns and population changes are relevant for equitable resource allocation. The cross sectional study design does not allow attribution of causality. Not being able to distinguish between types of diabetes was also one of the shortcomings of this study. In addition it is not possible to be certain whether all participants had fasted the night before. However, this study supports the need to identify risk factors, other than the traditional ones, which could explain the increasing number of people with diabetes in rural areas of LMIC in order to inform practice, policy and research. More recent data are required to monitor secular trends in prevalence of diabetes and its risk factors.

## Conclusion

Rwanda has rural-urban differences in diabetes prevalence with similar prevalence of impaired fasting glucose in both settings indicating the potential risk of increase of number of people with diabetes in the future if nothing is done to prevent diabetes. The scarce prevalence of traditional risk factors for hyperglycemia in the rural Rwandan population reveals the need for further research to understand the factors driving diabetes for evidence based and contextualized preventive strategies.

### What is known about this topic

- Diabetes prevalence is increasing with urbanization;
- Traditional factors, such as obesity, ageing, physical inactivity, high cholesterol and hypertension are reported to be associated with diabetes worldwide.

### What this study adds

- The prevalence of diabetes is higher in urban than in rural dwellers in Rwanda however, there is no significant rural-urban difference in the prevalence of impaired fasting glucose;
- The magnitude of the association with traditional risk factors for diabetes differ in rural and urban settings.
